# Comparative Proteomics Analysis of the Seedling Root Response of Drought-sensitive and Drought-tolerant Maize Varieties to Drought Stress

**DOI:** 10.3390/ijms20112793

**Published:** 2019-06-07

**Authors:** Wenjing Zeng, Yunling Peng, Xiaoqiang Zhao, Boyang Wu, Fenqi Chen, Bin Ren, Zelong Zhuang, Qiaohong Gao, Yongfu Ding

**Affiliations:** 1 College of Agronomy, Gansu Agricultural University, Lanzhou 730070, China; 18119493191@163.com (W.Z.); wuboyang555@163.com (B.W.); 18893115958@163.com (F.C.); 13909490549@163.com (B.R.); zhuangzl3314@163.com (Z.Z.); gaoqh3324@163.com (Q.G.); 18893810043@163.com (Y.D.); 2 Gansu Provincial Key Lab of Aridland Crop Science, Lanzhou 730070, China; zhaoxq3324@163.com

**Keywords:** maize, root system, drought stress, physiological response, proteomic analysis, iTRAQ, qRT-PCR

## Abstract

The growth and development of maize roots are closely related to drought tolerance. In order to clarify the molecular mechanisms of drought tolerance between different maize (*Zea mays* L.) varieties at the protein level, the isobaric tags for relative and absolute quantitation (iTRAQ) quantitative proteomics were used for the comparative analysis of protein expression in the seedling roots of the drought-tolerant Chang 7-2 and drought-sensitive TS141 maize varieties under 20% polyethylene glycol 6000 (PEG 6000)-simulated drought stress. We identified a total of 7723 differentially expressed proteins (DEPs), 1243 were significantly differentially expressed in Chang 7-2 following drought stress, 572 of which were up-regulated and 671 were down-regulated; 419 DEPs were identified in TS141, 172 of which were up-regulated and 247 were down-regulated. In Chang 7-2, the DEPs were associated with ribosome pathway, glycolysis/gluconeogenesis pathway, and amino sugar and nucleotide sugar metabolism. In TS141, the DEPs were associated with metabolic pathway, phenylpropanoid biosynthesis pathway, and starch and sucrose metabolism. Compared with TS141, the higher drought tolerance of Chang 7-2 root system was attributed to a stronger water retention capacity; the synergistic effect of antioxidant enzymes; the strengthen cell wall; the osmotic stabilization of plasma membrane proteins; the effectiveness of recycling amino acid; and an improvement in the degree of lignification. The common mechanisms of the drought stress response between the two varieties included: The promotion of enzymes in the glycolysis/gluconeogenesis pathway; cross-protection against the toxicity of aldehydes and ammonia; maintenance of the cell membrane stability. Based on the proteome sequencing information, the coding region sequences of eight DEP-related genes were analyzed at the mRNA level by quantitative real-time PCR (qRT-PCR). The findings of this study can inform the future breeding of drought-tolerant maize varieties.

## 1. Introduction

Drought is a major abiotic stress that limits agricultural productivity and results in significant crop yield losses worldwide. Maize (*Zea mays* L.) is the primary grain product and feed crop in China, but it is sensitive to drought [1], which is an important limiting factor for maize yield. Drought impacts the entire growth cycle at the seedling stage [2], maize exhibits several important physiological responses, including decreased cell turgor [3], inhibited CO_2_ exchange, and decreased photosynthetic efficiency and chlorophyll contents under water stress [4,5]. While the water requirements at the seedling stage are lower than during later stages of growth, drought stress will influence the adaptive ability of the plant at an early stage and limit the yield potential and growth of crops [6]. Therefore, elucidating the molecular mechanisms of the response of maize to drought stress at the seedling stage thus has great significance for maize production.

Proteins execute cellular functions that directly affect crop growth and development, the proteome is constantly changing along with the types, functions, physiological states, environmental conditions, and pathological states of the cells. Proteomics research is a core component of life science research in the post-genome era. It is used to compare the differences in protein expression of cells under different physiological or pathological conditions, to classify and identify related proteins, and to study protein interactions and protein functions. Previous studies on DEPs under stress in maize have relied on two-dimensional electrophoresis (2-DE), but proteins that are too large or small, too acidic or basic, too hydrophobic, or in low abundance are difficult to observe via 2D gel electrophoresis [7]. Proteomics is presently developing from qualitative to quantitative proteomics, and isobaric tags for relative and absolute quantitation (iTRAQ) is an accurate quantitative proteomics technique with high quantitative accuracy, high sensitivity, good repeatability, and high throughput [8].

Numerous studies exist on the proteomics of the drought stress response in the crop. For instance, Zenda et al. [6] studied the physiological and proteomic changes of the drought-tolerant YE8112 and drought-sensitive MO17 maize varieties under drought stress and attributed the higher drought-tolerance of YE8112 to the activation of photosynthetic proteins involved in balancing light capture and utilization; enhanced lipid metabolism; development of abiotic and biotic cross-tolerance mechanisms; and increased cellular detoxification capacity. Zhang et al. [9] analyzed the physiological responses and differentially expressed genes in the inbred line maize line B73 under water deficit and recovery conditions, and discovered that many genes that are differentially expressed in responses to drought stress and recovery affect photosynthetic systems and hormone biosynthesis. Zhang and Shi [10] studied the physiological and proteomic response of two alfalfa (*Medicago sativa* L.) varieties contrasting in drought tolerance and found that the stronger drought-tolerance of Longzhong was attributed to its higher osmotic adjustment capacity and its greater ability to orchestrate its enzymatic and non-enzymatic antioxidant systems, thus avoiding severe oxidative damage. Pan et al. [11] reported the proteomic changes in Italian ryegrass (*Lolium multiflorum*) under drought stress and found that drought-responsive proteins were closely related to metabolic processes, including signal transduction, antioxidant defenses, hydrolysis, and transmembrane transport. Cheng et al. [12] analyzed the proteomic changes of two wheat (*Triticum aestivum* L.) varieties with different drought tolerances subjected to dehydration treatments (18 h, 24 h, and 48 h) and rehydration treatment (24 h) using 2-DE, found that compared with Longchun 23, the resistance mechanisms of Xihan No.2 mainly included changes in the metabolism of carbohydrates and amino acids, the activation of more antioxidation and defense systems, as well as increase in the levels of proteins involved in ATP synthesis and protein degradation/refolding.

However, the majority of proteomic studies of crops under drought stress have focused on the shoots rather than the root system. Studies have shown that plant tolerance to adverse environmental conditions is highly correlated with the regulation of root development and stress response strategies [13]. In addition to physically supporting the plant, the maize root system absorbs large amounts of water and nutrients and thus has a great impact on maize yield and a close relationship with drought tolerance [14]. Studies have shown that root tolerance to drought stress depends on its ability to maintain adequate symplastic osmotic potential, cell wall protein composition, carbohydrate metabolism and the metabolic pathways involved in the oxidative stress response [15,16]. As maize roots grow underground, there are various difficulties associated with the sampling and analysis of root samples, and thus few proteomic analyses on the drought response of maize roots are available. Studies have shown that there are differences in the root characteristics among different drought-tolerant maize varieties [17], and thus root characteristics could be used as an important indicator of drought tolerance. Thus, elucidating the molecular mechanisms of the maize root response to drought stress is critical.

Therefore, in this study, we present a comprehensive comparative proteomic analysis of drought-tolerant Chang 7-2 and drought-sensitive TS141 maize seedling roots under 20% PEG 6000 stress using iTRAQ quantitative proteomics and bioinformatics analysis. Studying the proteomic changes of maize seedling roots under drought stress is of great significance for understanding the response mechanisms of root growth to drought stress. Our findings elucidate the differences in drought tolerance at the protein level between the roots of the two maize varieties and gain comprehensive knowledge of the underlying molecular mechanisms involved in drought stress. The findings will help drive further work, providing a foundation for the breeding and improvement of new drought-tolerant maize varieties.

## 2. Results

### 2.1. Phenotypic Differences between Chang 7-2 and TS141 Under PEG 6000 Stress

**Figure 1:**
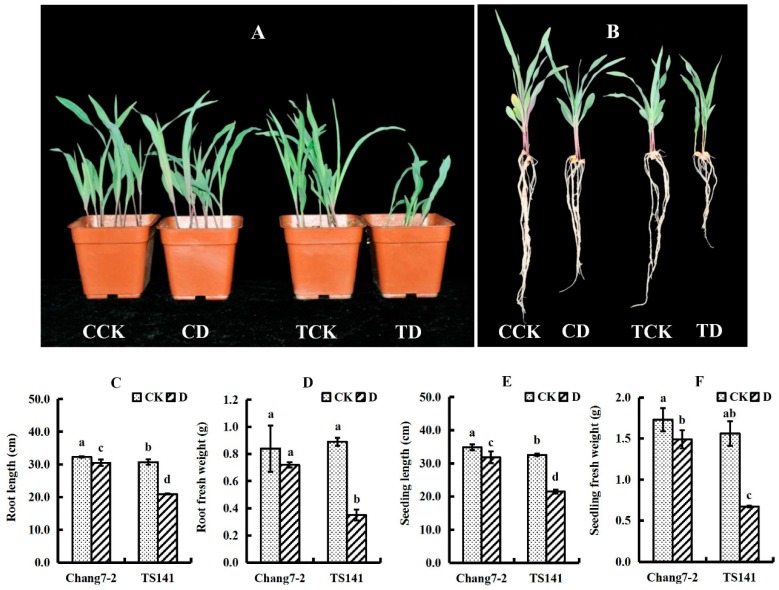
Growth parameters of Chang 7-2 and TS141 seedlings under control and drought-stressed conditions. Data are presented as mean ± standard deviation. Lowercase letters indicate a significant difference at the 1% level. CK: Control; D: Drought stress. CCK: Chang 7-2 control; CD: Chang 7-2 drought stress; TCK: TS141 control; TD: TS141 drought stress. (The same applies in the other tables and figures). (**A**,**B**) Performance of Chang 7-2 and TS141 seedlings after exposure to control or drought stress. (**C**) Root length (cm). (**D**) Root fresh weight (g). (**E**) Seedling length (cm). (**F**) Seedling fresh weight (g). *n* = 3.

Phenotypic differences were observed under 20% PEG-simulated drought stress. Under drought stress, Chang 7-2 grew more robustly than TS141 and did not differ from the control, while TS141 exhibited decreased plant height and severe root shortening (Figure 1A,B). Compared with the control, the two varieties displayed delayed growth under stressed conditions, but the decreases in seeding length (SL), root length (RL), seedling fresh weight (SFW), and root fresh weight (RFW) in TS141 were greater than in Chang 7-2 (Figure 1C–F).

### 2.2. Physiological Response of Chang7-2 and TS141 to PEG 6000 Stress

**Figure 2:**
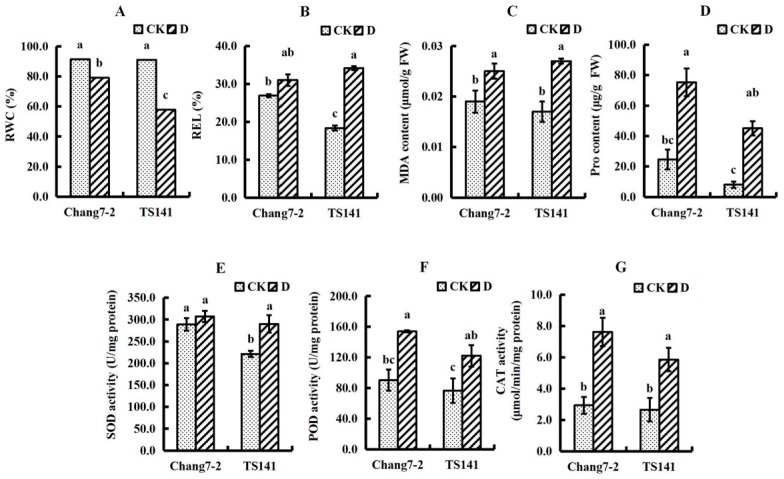
Physiological characteristics of Chang 7-2 and TS141 seedling roots under control and stressed conditions. Data are presented as the mean ± standard deviation. Lowercase letters indicate a significant difference at the 1% level. (**A**) RWC. (**B**) REL. (**C**) MDA content. (**D**) Pro content. (**E**) SOD activity. (**F**) POD activity. (**G**) CAT activity.

The relative water content (RWC), relative electrolyte leakage (REL), malondialdehyde (MDA), and proline (Pro) content, and superoxide dismutase (SOD), peroxidase (POD), and catalase (CAT) activities were measured in the seedling roots of the two varieties (Figure 2). The RWC of the root system in the two varieties reached 91% under control conditions, but decreased following drought treatment, and the decrease in TS141 was larger than that in Chang 7-2 (Figure 2A). The REL of TS141 increased following simulated drought stress, but was unchanged in Chang 7-2. (Figure 2B). The MDA content and Pro content of the two varieties increased following simulated drought stress, with the increase in MDA content of Chang 7-2 being less than that of TS141, while the increase in Pro content in Chang 7-2 was greater than that in TS141 (Figure 2C,D). In addition, the antioxidant enzyme activity in Chang 7-2 was higher than that in TS141 (Figure 2E–G). The activity of SOD increased in TS141 under stress conditions, but was unchanged in Chang 7-2, and the activity of POD and CAT increased under stress conditions in two varieties. The increase in POD activity and CAT activity in Chang 7-2 was greater than in TS141, whereas the change in SOD activity was not significantly different from the control.

### 2.3. Quantitative Proteomic Analysis

**Figure 3:**
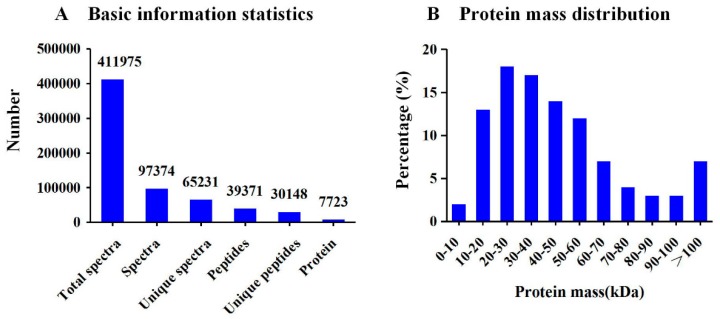
(**A**) Spectra, peptides, and proteins identified from isobaric tags for relative and absolute quantitation (iTRAQ) proteomics. (**B**) Molecular weight distribution of the proteins that were identified from the iTRAQ analysis.

**Figure 4:**
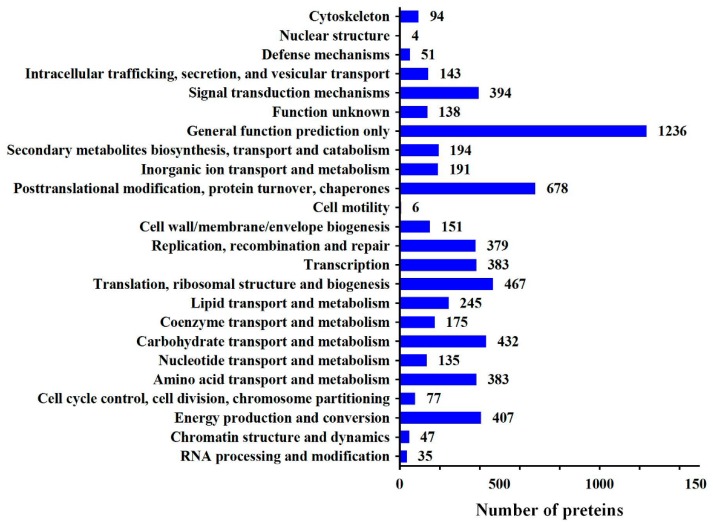
Cluster of Orthologous Groups of Proteins (COG) annotation analysis of all proteins.

A total of 7723 proteins were identified using iTRAQ in the maize seedling roots (Tables S1 and S2). We detected 97374 spectra (Table S3), 39371 peptides and 30148 unique peptides (Figure 3A). The identified protein molecular mass was mainly distributed between 10–60 kDa (Figure 3B). Cluster of Orthologous Groups of Proteins (COG) analysis divided these proteins into 24 categories, among which general function prediction contained the largest number of proteins (19.1%), followed by posttranslational modification, protein turnover, and chaperones (10.52%) (Figure 4).

### 2.4. iTRAQ Results

**Figure 5:**
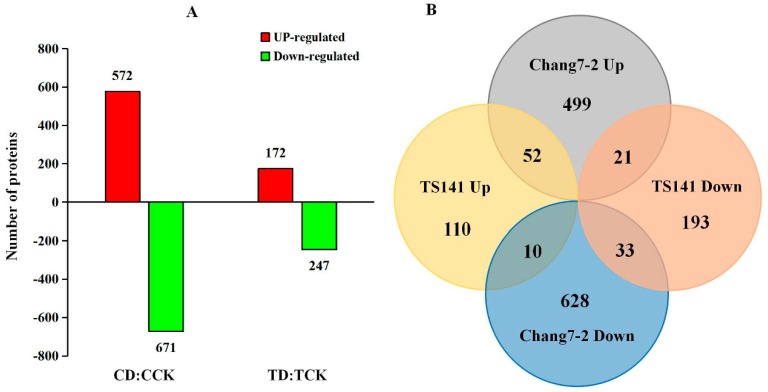
(**A**) Number of up-regulated and down-regulated proteins. (**B**) Venn diagram of the two maize varieties.

Based on the fold change of > 1.2 (up) or < 0.83 (down) and a *p*-value < 0.05, 1546 significant DEPs were identified. Compared with the control, there were 1243 DEPs in Chang 7-2 following stress treatment, 572 of which were up-regulated (Table S4) and 671 of which were down-regulated (Table S5). In TS141, 419 DEPs were detected following stress, 172 of which were up-regulated (Table S6) and 247 of which were down-regulated (Table S7, Figure 5A).

Among these proteins, 52 DEPs were commonly up-regulated in the two varieties (Table S8) and 33 DEPs were down-regulated. 21 proteins that were up-regulated in Chang 7-2 were down-regulated in TS141, and 10 proteins that were down-regulated in Chang 7-2 were up-regulated in TS141 (Table S9, Figure 5B).

### 2.5. Pathway Enrichment Analysis of DEPs

Pathway enrichment analysis was used to identify the most important biochemical metabolic processes that the DEPs were involved in. A pathway was considered to be significantly enriched at *p* < 0.05.

Compared with the control group, about 80.63% of the DEPs in the Chang 7-2 treatment group were distributed in 124 pathways. Ten pathways were considered to be significantly enriched (*p* < 0.05), including ribosome (54, 5.36%), glycolysis/gluconeogenesis (33, 3.28%), and amino sugar and nucleotide sugar metabolism (31, 3.08%) (Table 1).

Compared with the control group, about 82.35% of the DEPs in the TS141 treatment group were distributed in 105 pathways. Eleven pathways were considered to be significantly enriched *(p* < 0.05), including metabolic pathway (125, 35.71%), phenylpropanoid biosynthesis (18, 5.14%), and starch and sucrose metabolism (15, 4.29%) (Table 2).

### 2.6. qRT-PCR Analysis of DEPs

**Figure 6:**
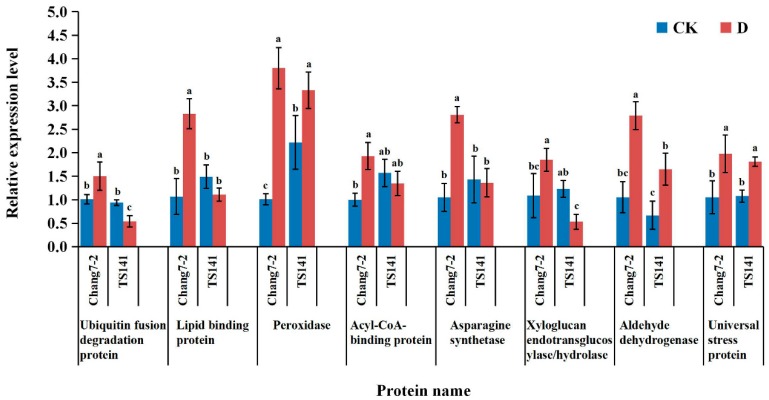
mRNA expression level analysis (qRT-PCR) of eight DEPs under control and stressed conditions. Data are presented as the mean ± standard deviation. Lowercase letters indicate a significant difference at the 5% level.

To confirm the reliability of the iTRAQ sequencing data, six proteins that were up-regulated in Chang 7-2 and down-regulated in TS141, and two proteins that were up-regulated in both varieties, were selected for qRT-PCR validation. The six proteins with different direction of accumulation included ubiquitin fusion degradation protein, lipid binding protein, POD, acyl-CoA-binding protein, asparagine synthetase, xyloglucan endotransglucosylase/hydrolase; the two proteins that were up-regulated in both varieties included aldehyde dehydrogenase and universal stress protein. According to the qRT-PCR results (Figure 6), the expression levels of the eight genes were increased significantly in drought-tolerance Chang 7-2, which was consistent with the results of the proteomic analysis. In contrast, the transcript levels of genes encoding ubiquitin fusion degradation protein, xyloglucan endotransglucosylase/hydrolase, aldehyde dehydrogenase and universal stress protein were consistent with the results of the proteomic analysis in drought-sensitive TS141, while the expression levels of lipid binding protein, acyl-CoA-binding protein, and asparagine synthetase were unchanged. However, the expression level of POD differed, as it increased in the two varieties. This difference may be due to various post-transcriptional regulation and post-translational modification processes following drought stress. Overall, the observed transcription patterns of these genes were consistent with the results of the proteomic analysis.

## 3. Discussion

Maize is subjected to a variety of biotic and abiotic stresses during growth and development. Of these stresses, drought stress is one of the major abiotic stresses affecting germination and the seedling stage of maize in particular. Screening for drought-tolerant varieties and research into the drought tolerance mechanisms have significance for maize production. In this study, iTRAQ was used to identify the root proteins of the drought-tolerant maize inbred line Chang 7-2 and drought-sensitive maize inbred line TS141 at the seedling stage under drought stress.

### 3.1. Changes in Growth Parameters and Physiological Indicators

The growth parameters and physiological traits indicated that these two maize inbred lines performed differently under drought stress conditions.

Compared with control conditions, the drought-treated seedlings of both varieties exhibited retarded growth. SL, RL, SFW, and RFW decreased significantly in the roots of the two inbred lines under simulated drought stress. Additionally, the decreases in SL, RL, SFW and RFW were greater in TS141 than in Chang 7-2 (Figure 1C–F).

RWC decreased significantly in the roots of both inbred lines under stressed conditions, and the decrease in RWC in Chang 7-2 (13.5%) was less than in TS141 (36.5%). RWC was generally higher in the Chang 7-2 seedling roots both under control and stressed conditions (Figure 2A). Studies found that RWC was significantly higher in the tolerant maize genotype than the sensitive genotype under control and drought conditions [18]. It is thus speculated that the higher RWC of Chang 7-2 allows it to maintain more effective physiological and biochemical processes under drought stress.

The level of REL reflects the degree of damage to the plant cell membrane under osmotic stress. Under drought stress, the smaller the REL, the stronger the drought tolerance. MDA is the final decomposition product of membrane lipid peroxidation, and the rise in MDA content under stress conditions suggests that drought stress could induce membrane lipid peroxidation as a result of increased reactive oxygen species (ROS) [19], and its content directly reflects the extent of damage to the membrane system. Pro exists in the cytoplasm of plants. Studies have shown that a higher Pro content can effectively reduce the higher osmotic potential caused by osmotic stress in plants, protect the stability of the cell membrane system, and prevent cell dehydration [20]. In our study, the MDA content and Pro content of the two varieties increased under stressed conditions, indicating that the membrane systems of the two varieties were damaged under drought stress. The REL and MDA content were higher in Chang 7-2 than in TS141 under control conditions, but under stressed conditions, the REL and MDA content were higher in TS141 and the increase in MDA content in TS141 was greater than in Chang 7-2. These findings suggest that the osmotic stress caused by drought on the membrane system of Chang 7-2 was relatively low. Additionally, the Pro content in Chang 7-2 was also significantly increased, indicating higher cell homeostasis in Chang 7-2 than in TS141 (Figure 2B–D). We suggest that the high stability of the cell membrane in Chang 7-2 root system provide a normal cellular environment to cope with drought stress.

SOD, POD, and CAT are important antioxidant enzymes in plants that eliminate the ROS and peroxides induced by stress, inhibit the peroxidation of the plasma membrane, and protect cells from damage [21]. Under stressed conditions, the activity of these enzymes increased, and the activity in Chang 7-2 was higher both under control and stressed conditions. The increase in POD activity and CAT activity in Chang 7-2 was greater than in TS141. Although the SOD activity in Chang 7-2 unchanged under stress condition, its activity was still higher than in TS141 (Figure 2E–G). Studies have shown that the increase in the activity of these enzymes under stress conditions protects plant cells from the oxidative damage emanating from the ROS generated under such conditions [18]. We suggest that Chang 7-2 can effectively eliminate free radicals under simulated drought stress.

From the results of the physiological experiments, it can be inferred that the maize seedling roots exhibit stress tolerance mechanisms under simulated drought stress. The high RWC and Pro content both under the control and stressed conditions, the higher increase in SOD, POD, and CAT content, and the lower REL and MDA content after stress conferred Chang 7-2 with stronger drought resistance. The stronger water retention capacity, the ability to maintain plasma membrane balance, and the effectiveness in eradicating ROS made Chang 7-2 more resistant to drought.

### 3.2. The Drought Response of Drought-Tolerance Inbred Line Chang 7-2

Among the DEPs, it was found that following drought stress treatment, 31 proteins exhibited a different direction of accumulation in the two varieties, and these proteins accounted for the increased drought resistance of Chang 7-2 (Table S9).

#### 3.2.1. Antioxidant-related proteins are the major Drought Tolerance Signature in Chang 7-2

Adverse stress, such as drought, usually results in the excessive accumulation of ROS in plant cells, which eventually leads to oxidative stress. POD is as an important antioxidant enzyme for organisms and acts synergistically with SOD, CAT, and glutathione peroxidase to eliminate harmful free radicals under adverse conditions in a variety of crops [22,23,24]. In our study, it was found that peroxidase (tr|A0A1D6MRI4|A0A1D6MRI4_MAIZE, tr|B4FVT1|B4FVT1_MAIZE) was significantly up-regulated in Chang 7-2 and down-regulated in TS141 under stressed conditions. Furthermore, in all of the selected DEPs, there were five POD points that were up-regulated in the roots of Chang 7-2, but only one in TS141. Our results demonstrated that maize seedling roots also possess a ROS scavenging system that responds to drought stress. Chang 7-2 accumulates more antioxidant enzymes, which can more effectively eliminate ROS. This is consistent with the physiological results of POD.

#### 3.2.2. Increasing Lignin Content is Effective to Resist Drought Stress in Chang 7-2 Root System

**Figure 7:**
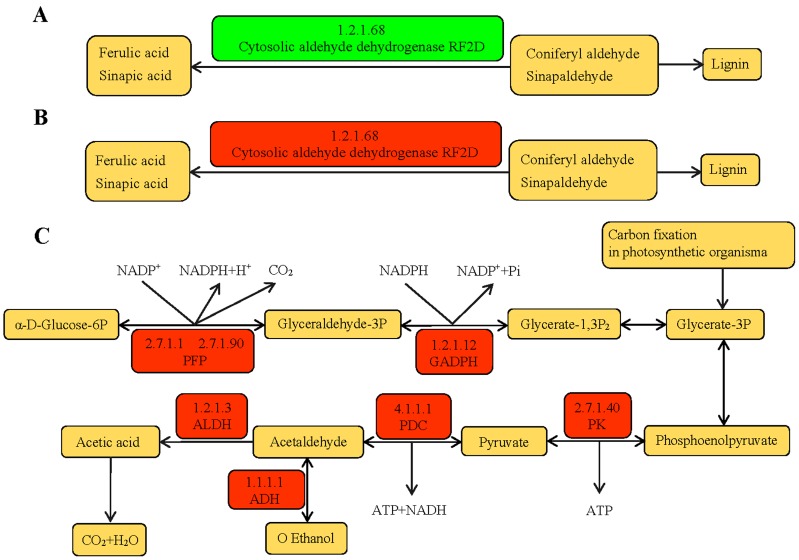
(**A**) Glycolysis/gluconeogenesis pathway. (**B**) Phenylpropanoid biosynthesis pathway in CD vs. CCK. (**C**) Phenylpropanoid biosynthesis pathway in TD vs. TCK. Red boxes represent the up-regulated proteins under simulated drought stress. Green boxes represent the down-regulated proteins under simulated drought stress. Yellow boxes represent metabolites.

The DEPs of TS141 were more enriched in phenylpropanoid biosynthesis (18, 5.14%) under drought stress. Although this pathway was not screened in Chang 7-2, it was found that the spots of 1.2.1.68 exhibited differences in the two varieties (Figure S1). At 1.2.1.68, cytosolic aldehyde dehydrogenase RF2D (tr|Q8S529|Q8S529_MAIZE) was down-regulated in Chang 7-2 and up-regulated in TS141. Under control conditions, the expression level of cytosolic aldehyde dehydrogenase RF2D in Chang 7-2 seedling roots was higher than in TS141. However, under simulated drought stress, this protein was up-regulated in TS141 with an expression multiple of 0.3, but was down-regulated in Chang 7-2 with an expression multiple of 0.4. In the phenylpropanoid biosynthesis pathway, low expression of cytosolic aldehyde dehydrogenase RF2D prevents more coniferyl aldehyde (CA) and sinapaldehyde (SA) from being converted into ferulic acid and sinapic acid. CA and SA are precursors of lignin, suggesting that the roots of Chang 7-2 respond to drought stress by producing more lignin. Studies have shown that lignin is the main component of the cell wall, participating in plant defense against fungi and enhancing the resistance to lodging and drought. Increased lignin content during drought stress can maintain the normal osmotic pressure of the cells, thereby enhancing the drought resistance of plants [25] (Figure 7A,B).

#### 3.2.3. Up-Regulation of Plasma Membrane Proteins could Effectively Relieve the Leaking of Cell Contents in Chang 7-2

Lipids not only provide energy for plant growth, but also participate in physiological and pathological reactions in plants, particularly in stabilizing cell membranes and recognizing and responding to biological abiotic stresses, such as drought tolerance [26]. The acyl-CoA-binding protein is a class of acyl carrier protein that plays an important role in the synthesis and transport of lipids. Studies have indicated that acyl-CoA-binding protein family affect the responses of plants to biological and abiotic stresses, including heavy metal oxidation, drought, low temperature, and pathogen infestation [27]. In our study, these proteins only contained two lipid-related proteins, one of which was the acyl-CoA-binding protein (tr|B4FER8|B4FER8_MAIZE), which was significantly up-regulated in Chang 7-2 and down-regulated in TS141. GO analysis showed that, in a biological process, it participated in the lipid transport process; and in a cellular component, it participated in a plasma membrane and cytosol processes. It is speculated that compared with TS141, Chang 7-2 could enhance the stability of the cell membrane by increasing the expression of an acyl-CoA-binding protein, thus alleviating the damage of drought stress. The expression level of another lipid-related protein, namely lipid binding protein (tr|B6ST81|B6ST81_MAIZE), was also significantly up-regulated in Chang 7-2, but down-regulated in TS141. It is inferred that compared with TS141, Chang 7-2 can more effectively reduce damage to the cell membrane under drought conditions and relieve the leaking of cell contents, thereby contributing to improved drought resistance in Chang 7-2. This is consistent with the physiological results of REL, MDA and Pro. In our study, the expression of asparagine synthetase (tr|B5U8J8|B5U8J8_MAIZE) was up-regulated in Chang 7-2 and down-regulated in TS141 under stressed conditions. Studies have shown that asparagine may have a function similar to Pro-regulated cell infiltration [28], as osmotic stress increased the expression of the asparagine synthetase gene in wheat roots [29]. Here, we speculated that Chang 7-2 maintains the nitrogen metabolism balance by increasing asparagine synthetase to improve drought tolerance, and at the same time, the high expression of asparagine synthetase also plays a role in balancing the normal osmotic pressure of the cells. However, the specific role of the maize roots in inducing asparagine synthetase gene expression under drought stress is not fully understood, and the expression change of asparagine synthetase found in this study may provide a foundation for future research.

#### 3.2.4. Chang 7-2 could Tolerates Drought Stress by Strengthening the Cell Wall and Elongating the Roots

Xyloglucan endotransglucosylase/hydrolase is a key enzyme in the process of plant cell wall remodeling that functions in strengthening the cell wall and maintaining the integrity of the cell wall [30]. We discovered that xyloglucan endotransglucosylase/hydrolase (tr|Q42446|Q42446_MAIZE) was significantly up-regulated in Chang 7-2 and down-regulated in TS141 under stressed conditions. It is speculated that compared with TS141, Chang 7-2 has a stronger ability to maintain cell wall integrity under drought stress, thereby maintaining a stable cellular environment. In addition, xyloglucan endotransglucosylase/hydrolase plays an important role in root elongation [31], which suggests that the root system of Chang 7-2 has a stronger ability to extend deep into the soil, thus obtaining water from lower depths. Studies have shown that its overexpression in *A. thaliana* can increase the drought tolerance of transgenic plants [32]. It is speculated that Chang 7-2 tolerates drought stress by strengthening the cell wall and elongating the roots. To date, no studies have reported on the expression level changes and roles of xyloglucan endotransglucosylase/hydrolase in maize seedling roots under drought stress, and thus the findings of this study could provide reference data for future research on the mechanisms of drought tolerance in maize.

#### 3.2.5. Chang 7-2 is More Efficient in Recycling Amino Acids from Proteins that have been Inactivated by Drought Stress.

Ubiquitin fusion degradation protein is a key regulator in protein degradation via the ubiquitin 26S proteasome pathway. In our study, the expression level of an ubiquitin fusion degradation protein (tr|A0A1D6DSN6|A0A1D6DSN6_MAIZE) was up-regulated in Chang 7-2 and down-regulated in TS141. Studies have indicated increased expression of ubiquitin fusion degradation protein in *A. thaliana* [33] under water stress, which suggested Chang 7-2 was more efficient in recycling amino acids from proteins that have been inactivated by drought exposure.

### 3.3. The Commonality Metabolic Changes of Two Varieties

Among the DEPs, it was found that following drought stress treatment, 52 proteins were up-regulated in the two varieties, and these proteins reflected the commonality of metabolic changes in resistance to drought stress (Table S8).

#### 3.3.1. Proteins Involved in Cell Detoxification under Drought Stress

ROS can promote the peroxidation of membrane lipids, leading to the accumulation of aldehydes in the organism and damage to the cells [34]. Aldehyde dehydrogenase can catalyze the oxidative dehydrogenation of endogenous or exogenous aldehydes to generate corresponding carboxylic acids by combining NAD^+^ or NADP^+^ [35] to reduce the accumulation of aldehydes in plants and achieve their detoxification purposes. Studies have found that aldehyde dehydrogenase is up-regulated to reduce the toxicity of aldehydes under drought stress [36]. In addition, alcohol dehydrogenase is also essential in this metabolic activity. Alcohol dehydrogenase and aldehyde dehydrogenase are involved in the catalytic decomposition of ethanol, which is eventually oxidized into CO_2_ and H_2_O, providing an intermediate product for plant metabolism. In addition, acetaldehyde can also produce ethanol through the reverse reaction of alcohol dehydrogenase, thus avoiding the toxicity of acetaldehydes. In Chang 7-2 and TS141, aldehyde dehydrogenase (tr|Q7FWR0|Q7FWR0_MAIZE) and alcohol dehydrogenase (tr|B6TD57|B6TD57_MAIZE, sp|P00333|ADH1_MAIZE, tr|B6SUD1|B6SUD1_MAIZE) were up-regulated under stress conditions, indicating that these proteins participate play an important role in eliminating the toxic effects of aldehydes.

Additionally, it was found that glutamate synthase (tr|A0A1D6NFL0|A0A1D6NFL0_MAIZE, tr|A0A1D6G281|A0A1D6G281_MAIZE) was also up-regulated in two varieties. Ammonia produced by reduction of NO_3_^−^ or NO_2_^−^ is toxic if it is accumulated in plant cells [37], and the up-regulation of glutamate synthase leads to an increase in the abundance of glutamate, which can be used for maize growth [38], and for eliminating ammonia produced under adverse conditions [39,40].

#### 3.3.2. The Glycolysis/Gluconeogenesis is the Major Pathway for Maize Roots to Cope with Drought Stress

Pathway enrichment analysis showed that the glycolysis/gluconeogenesis pathway was screened in the two varieties. In Chang 7-2, the number of DEPs in this pathway was particularly large (33, 3.28%). The glycolysis/gluconeogenesis pathway responds to biotic and abiotic stress mainly by affecting the ATP supply to plants. Comparing the two groups of the pathway, we found that pyrophosphate: Fructose-6-phosphate 1-phosphotransferase (PFP) (2.7.1.90, 2.7.1.11), Glyceraldehyde 3-phosphate dehydrogenase (GAPDH) (1.2.1.12), PK (2.7.1.40), pyruvate decarboxylase (PDC) (4.1.1.1), aldehyde dehydrogenase (1.2.1.3), and alcohol dehydrogenase (1.1.1.1) were increased in the two varieties (Figure 7C), resulting in the abundant production of ATP and NADPH.

PFP catalyzes the interconversion between fructose-6-phosphate and fructose-1, 6-bisphosphate. This process is catalyzed by phosphofructokinase (PFK) in most cases, and the difference is that the reaction catalyzed by PFK is irreversible and requires ATP, while the reaction catalyzed by PFP is reversible and ATP is replaced by pyrophosphoric acid (PPi). Studies have shown that the parallel effects of PFK and PFP can enhance the rate of glycolysis and the production of ATP under hypoxic conditions [41]. In our study, PFP (tr|B7ZYR6|B7ZYR6_MAIZE, tr|A0A1D6MAI0|A0A1D6MAI0_MAIZE) was significantly up-regulated in the two varieties. The increase in PFP expression may be an adaptive mechanism for maize roots to cope with drought stress through glycolysis. In addition, it was found that the expression level of a fructokinase-2 (tr|B6TB29|B6TB29_MAIZE) was also increased in the two varieties. This protein is an allosteric activation enzyme of PFP, and the increase in fructokinase-2 expression also promotes the generation of energy by cells through the glycolysis pathway. Pyruvate kinase (PK) is one of the main rate-limiting enzymes in glycolysis. It catalyzes the final step of glycolysis, converting phosphoenolpyruvate and ADP into ATP and pyruvate acid. Pyruvic acid also generates ATP through the TCA cycle. Under drought stress, this protein (tr|B4F9G8|B4F9G8_MAIZE) was significantly up-regulated in the two varieties, and it is presumed that the increase in PK expression may have produced more energy via the glycolysis pathway to tolerate drought.

GAPDH is an essential enzyme in glycolysis and gluconeogenesis [42]. In our study, the expression of GAPDH (sp|Q43247|G3PC3_MAIZE, tr|Q6LBU9|Q6LBU9_MAIZE) was significantly up-regulated in the two varieties under stressed conditions, and the expression of GAPDH increased more in Chang 7-2. Studies have shown that the overexpression of the GAPDH gene in rice controlled the excessive accumulation of H_2_O_2_ and alleviated oxidative stress [9]. We suggest that GAPDH alleviates ROS-induced cell damage in maize under drought stress. In addition, the outputs and cycles of the Calvin cycle photosynthetic products are related to the activity of GAPDH, and it is speculated that under drought stress, maize roots would increase the expression of GAPDH after assimilating CO_2_, which promotes the outward transfer of intermediate products, thereby providing more raw materials for other synthetic reactions, as suggested by Rochat et al. [43].

The accumulation of pyruvate in the glycolysis pathway has negative impacts, and PDC controls the anaerobic fermentation of pyruvate. In our study, the expression levels of PDC (tr|Q8S4W8|Q8S4W8_MAIZE, tr|C4J495|C4J495_MAIZE) were up-regulated in the two varieties under stressed conditions, and the expression level in Chang 7-2 increased more, suggesting that the increase in PDC in maize roots under drought stress not only ensures the continuous process of glycolysis, but also consumes the NADH produced during glycolysis such that the cells are not damaged by acidification. Current studies on maize only indicate the up-regulation of PDC expression following waterlogging in seedlings [44]. The changes in PDC under other environmental stresses remain unclear.

#### 3.3.3. Maintain Cell Membrane Stability is A Common Strategy for Maize Roots under Drought Stress

S-adenosylhomocysteine hydrolase is a key enzyme that widely exists in organisms and regulates intracellular methylation [45]. Weretilnyk et al. [46] found that the expression of s-adenosylhomocysteine hydrolase doubled in betaine accumulated plants under salt stress. Li et al. [47] found that the expression of the s-adenosylhomocysteine hydrolase gene gradually increased as the degree of drought increased in *Kalanchoe daigremontiana*; the lipid peroxidation degree of transgenic *K. daigremontiana* s-adenosylhomocysteine hydrolase tobacco was relatively mild under drought stress, and the tobacco could maintain relatively stable metabolic functioning. These facts indicate that s-adenosylhomocysteine hydrolase may play a role in protecting the plasma membrane of the cells. In our study, the expression level of this protein (tr|B4G0V3|B4G0V3_MAIZE) was up-regulated in the two varieties under stressed conditions, and the range of the increase was particularly evident in Chang 7-2. Therefore, it is speculated that maize roots could slow down cell membrane damage via the up-regulation of s-adenosylhomocysteine hydrolase, thereby improving drought tolerance.

Non-symbiotic hemoglobin exists in many monocotyledonous and dicotyledonous plants [48]. Studies have indicated that the expression of non-symbiotic hemoglobin is weak under normal conditions, while the expression of non-symbiotic hemoglobin increases under nutritional deficiency, bacterial infection, and hormone treatment [49,50,51]. In our study, the non-symbiotic hemoglobin (tr|B4FSM7|B4FSM7_MAIZE) was up-regulated in the two varieties. Thus, we propose that maize may produce more non-symbiotic hemoglobin in order to adapt to drought stress. In addition, non-symbiotic hemoglobin also exhibits POD-like activity and participates in nitrous oxide metabolism, and the coordinated induction of non-symbiotic hemoglobin could finely regulate the nitrous oxide steady state and protect maize roots from nitrification [52]. Recombinant *A. thaliana* non-symbiotic hemoglobin AtGLB1, AtGLB2, and AtGLB3 showed that they could rely on H_2_O_2_ to oxidize some substrates of peroxide, indicating POD-like activity [53]. In our study, GO analysis showed that this protein participated in the cell wall and plasma membrane processes in the cell component items. Therefore, it is speculated that non-symbiotic hemoglobin may play a role in maintaining cell membrane stability in maize roots under drought stress.

#### 3.3.4. Enhancement of Ethylene May Contribute to Enhance Drought Tolerance of Maize Roots

1-Amino-cyclopropane-1-carboxylate oxidase (ACO) catalyzes the final step of ethylene biosynthesis, in which the precursor 1-aminocyclopropane-1-carboxylate (ACC) is converted to ethylene. In our study, this protein (tr|C0PEP2|C0PEP2_MAIZE) was up-regulated in the two varieties, and the range of increase was particularly evident in Chang 7-2. Gupta et al. [54] suggested that plants promote increased ethylene production by increasing ACC synthesis and its conversion to ethylene under drought stress, and ethylene application could enhance drought tolerance [55]. However, some reports are inconsistent with our results; for instance, Chen et al. [56] found that TaACO1 mRNA was down-regulated by salt, drought, oxidative stress, and ABA in *A. thaliana*. This may be due to the different regulatory mechanisms of the different research materials, or could be that the response of plants to drought stress is a dynamic process, and most experiments, including our study, are only performed at one or several time nodes, resulting in inconsistent conclusions.

#### 3.3.5. Up-Regulation of Alanine Aminotransferase (AlaAT) could Maintain the respiration and Carbon-Nitrogen Balance in Maize Roots

AlaAT is located in the mitochondria of plants, such as *A. thaliana* [57]. The location of AlaAT in the mitochondria may be directly related to its metabolic function [58], as mitochondrial AlaAT seems to be able to activate mitochondrial alternative oxidase by increasing the content of pyruvate, thereby regulating plant respiratory oxygen consumption [59]. In our study, the expression level of this protein (tr|O82443|O82443_MAIZE, tr|A0A1D6KCZ2|A0A1D6KCZ2_MAIZE) was up-regulated in the two varieties under stressed conditions, and compared with TS141, the expression of AlaAT was more greatly up-regulated in Chang 7-2. GO analysis showed that this protein participated in mitochondria-related biological processes in cellular components, which is consistent with previous studies. Rocha et al. [60] found that the AlaAT gene was most highly expressed in the roots and nodules, and thus it is speculated that the function of the AlaAT gene may be more dependent on the roots. Therefore, we speculated that AlaAT may play an important role in the transport of pyruvate or alanine after drought stress in maize roots, which is conducive to maintaining the respiration and carbon-nitrogen balance of the whole plant.

### 3.4. Proposed Molecular Model of the Response of Chang7-2 to Drought Stress

**Figure 8:**
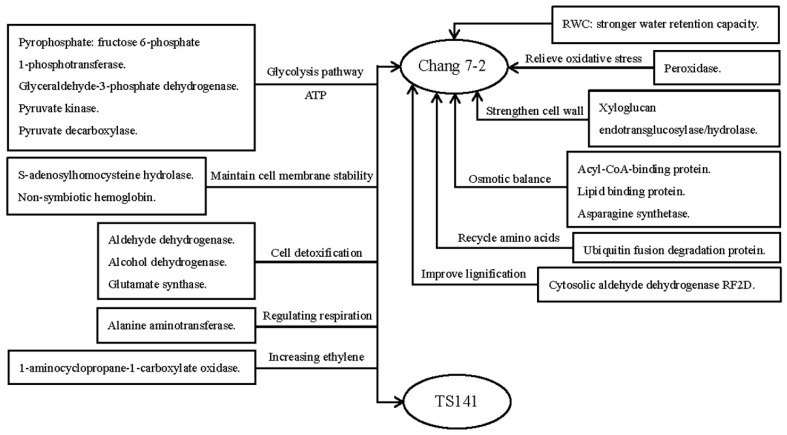
Molecular model of the response of Chang 7-2 to drought stress.

Based on the analysis above, we propose a molecular model for the response of Chang 7-2 to simulated drought stress (Figure 8). The mechanism of Chang 7-2 in response to drought is attributed to: The stronger water retention capacity; the synergistic action of antioxidant enzymes, such as POD, which eliminates harmful free radicals and alleviates oxidative stress; strengthening the cell wall through the up-regulation of xyloglucan endotransglucosylase/hydrolase; the effects of acyl-CoA-binding protein, lipid binding protein, and asparagine synthetase, which can relieve the extravasation of cellular contents, maintain osmotic balance, and stabilize the membrane system of plants. Besides, the increase in ubiquitin fusion degradation protein makes Chang 7-2 more efficient in recycling amino acids from proteins that have been inactivated by drought exposure. A reduction in cytosolic aldehyde dehydrogenase RF2D improves lignification. Furthermore, the two varieties exhibited a common mechanism under drought stress: More energy was produced through the glycolysis/gluconeogenesis pathway and related enzymes, and aldehyde dehydrogenase, alcohol dehydrogenase and glutamate synthase alleviated the toxic effects of the aldehydes and ammonia; maintain cell membrane stability through the effective of s-adenosylhomocysteine and non-symbiotic hemoglobin. Some enzymes also responded to drought stress by regulating respiration and increasing the ethylene content.

## 4. Materials and Methods

### 4.1. Plant Materials and Drought Treatment

The drought-tolerant inbred line Chang 7-2 and drought-sensitive inbred line TS141 were used in our experiment. Using 20% PEG to simulate drought stress. Seeds were surface sterilized with 0.5% NaClO for 10 min and washed five times with sterile distilled water. The seeds were soaked in 20% PEG 6000 under dark conditions for 48 h at 25 °C and germinated on a double layer of sterilized filter paper. Germinated seeds were then plated in pots (size: 15 cm × 13 cm; 10 seedlings per pot). The greenhouse conditions were controlled at 25/20 °C day/night with a photoperiod of 16/8 light/dark and 60% relative humidity. Fifty milliliters of distilled water was added every other day, and 50 mL of 20% PEG 6000 was applied every three days. The control was supplied with distilled water only. Seedlings at the three-leaf stage were selected to determine growth parameters, and the roots were used to evaluate physiological traits. Three biological replicates were set for each treatment. Other parts of the seedling roots were immediately frozen in liquid nitrogen and stored at −80 °C cryogenic refrigerator for the protein extraction and proteome analysis. Two biological replicates were set for each treatment.

### 4.2. Determination of Growth Parameters and Physiological Indicators

The SL, RL, SFW, and RFW were measured at the three-leaf stage. The RWC was estimated according to Galmés et al. [61], REL was measured as described by Liu et al. [62], MDA content determination used the thiobarbituric acid reaction as described by Madhava Rao and Sresty [63], Pro content was quantified according to a ninhydrin-based colorimetric assay [64], SOD activity was determined using the nitroblue tetrazolium photoreduction method [65], POD activity was estimated by the guaiacol method [66], and CAT activity was determined as described by Siminis et al. [67].

### 4.3. Protein Extraction

iTRAQ analysis was implemented at BGI (Shenzhen, China). The extraction of protein was carried out according to the company’s method [68,69]. Samples weighing 2 g were placed into a mortar to which 10% PVPP was added. The samples were then ground in liquid nitrogen, and Lysis Buffer (7 M urea, 2 M thiourea, 4% CHAPS, 40 mM Tris-HCL; pH 8.5), 1 mM PSMF, and 2 mM EDTA were added and the samples were placed in an ultrasonic ice bath for 5 min. Afterward, 10 mM DTT was added and the mixture was sonicated for 5 min in an ice bath, followed by centrifugation at 15,000 rpm for 20 min at 4 °C. The supernatant was transferred to a new centrifuge tube to which 10% TCA and 10 mM DTT were added at 5 times the volume of the supernatant, and the solution was precipitated for 2 h at −20 °C. The samples were then centrifuged at 15,000 rpm for 20 min at 4 °C and the supernatant was discarded. The sediment was transferred into a new tube to which 1 mL TCA and 10 mM DTT were added. After being ground, the sediment was placed at -20 °C for approximately 30 min, centrifuged at 25,000 rpm for 20 min at 4 °C, and the supernatant was discarded. This process was repeated several times until the supernatant became colorless. The precipitate was air-dried and the appropriate amount of Lysis Buffer, 1 mM PSMF, and 2 mM EDTA were added and the samples were placed in an ultrasonic ice bath for 5 min, after which 10 mM DTT was added and the mixture was sonicated for 5 min in an ice bath and then centrifuged at 25,000 rpm for 20 min at 4 °C. The supernatant was transferred to a new tube to which 10 mM DTT was added, and the tube was placed in a water bath at 56 °C for 1 h. Upon returning to room temperature, 55 mM IAA was added, and the tube was kept in the dark for 45 min. The samples were precipitated in five times the volume of cold acetone for 2 h and then centrifuged at 25,000 rpm for 20 min and 4 °C, after which the supernatant was discarded. The appropriate amount of Lysis Buffer was added, and the sample was placed in a sonicating ice bath for 5 min and centrifuged at 25,000 rpm for 20 min at 4 °C, and then the supernatant was collected. The protein concentration was determined using the Bradford dye-binding assay [70].

### 4.4. Protein Digestion and iTRAQ Quantification

The protein solution (100 μg) from each sample was digested with Trypsin Gold (Promega, Madison, WI, USA) with the ratio of protein: Trypsin = 40:1 at 37 °C for 4 h. Then, Trypsin Gold was added once again with a ratio of protein: Trypsin = 40:1, and digested for 8 h at 37 °C. After trypsin digestion, peptides were desalted with a Strata X C18 column (Phenomenex, Torrance, CA, USA) and vacuum-dried. The peptides were dissolved in 0.5 M TEAB with vortexing. After the iTRAQ labeling reagents reached ambient temperature, they were transferred and combined with appropriate samples. The Chang 7-2 control groups were labeled with iTRAQ115 and 116; the Chang 7-2 treatment groups were labeled with iTRAQ113 and 114; the TS141 control groups were labeled with iTRAQ119 and 121; and the TS141 treatment groups were labeled with iTRAQ117 and 118. The labeled peptides with different reagents were combined and desalted with a Strata X C18 column (Phenomenex, Torrance, CA, USA) and vacuum-dried according to the manufacturer’s protocol.

### 4.5. Peptide Fractionation and LC-ESI-MS/MS Analysis

The peptides were separated on a Shimadzu LC-20AB HPLC Pump system coupled with a high pH RP column. The peptides were reconstituted with buffer A (5% ACN, 95% H_2_O, adjust pH to 9.8 with ammonia) to 2 mL and loaded onto a column containing 5-μm particles (Phenomenex). The peptides are separated at a flow rate of 1 mL/min with a gradient of 5% buffer B (5% H_2_O, 95% ACN, adjust pH to 9.8 with ammonia) for 10 min, 5–35% buffer B for 40min, and 35–95% buffer B for 1 min. The system is then maintained in 95% buffer B for 3 min and decreased to 5% within 1 min before equilibrating with 5% buffer B for 10 min. Elution was monitored by measuring absorbance at 214 nm, and fractions were collected every 1 min. The eluted peptides are pooled as 20 fractions and vacuum-dried.

Each fraction was resuspended in buffer A (2% ACN and 0.1% FA in water) and centrifuged at 20,000 rpm for 10 min. The supernatant was loaded onto a C18 trap column with a mobile phase flow rate at 5 μL/min for 8 min using an LC-20AD nano-HPLC instrument (Shimadzu, Kyoto, Japan). Then, the peptides were eluted from the trap column and separated on an analytical C18 column (inner diameter 75 μm) packed in-house. The gradient was run at 300 nL/min starting from 8 to 35% of buffer B (2% H_2_O and 0.1% FA in ACN) for 35 min, then going up to 60% for 5 min, then maintained at 80% B for 5 min, and finally returned to 5% in 0.1 min and equilibrated for 10 min.

Data acquisition was performed with a TripleTOF 5600 System (SCIEX, Framingham, MA, USA) equipped with a Nanospray III source (SCIEX, Framingham, MA, USA), a pulled quartz tip as the emitter (New Objectives, Woburn, MA, USA) and controlled with software Analyst 1.6 (AB SCIEX, Concord, ON, USA). Data were acquired with the following MS conditions: Ion spray voltage 2300 V, curtain gas of 30, nebulizer gas of 15, and interface heater temperature of 150 °C. High sensitivity mode was used for the whole data acquisition. The accumulation time for MS1 was 250ms, and the mass ranges were from 350 to 1500 Da. Based on the intensity in MS1 survey, as many as 30 product ion scans were collected exceeding a threshold of 120 counts per second (counts/s) and with charge-state 2+ to 5+, dynamic exclusion was set for 1/2 of peak width (12 s). For iTRAQ data acquisition, the collision energy was adjusted to all precursor ions for collision-induced dissociation and the Q2 transmission window for 100 Da was 100%.

### 4.6. qRT-PCR

Total RNA was extracted from snap-frozen root samples using TRIzol reagent (Tiangen Biotech, Beijing, China), and the RNA was reverse-transcribed using the FastKing RT Kit (with gDNase) (Tiangen Biotech). The qRT-PCR used the TB Green TM Premix Ex Taq ^TM^ II kit (TaKaRa, Dalian, China) in the following 20-μL reaction system: 2 μL cDNA (50 ng/μL), 0.8 μL forward primer (10 μmol/L), 0.8 μL reverse primer (10 μmol/L), 10 μL TB Green Premix Ex Taq II (2×), 0.4 μL ROX Reference Dye (50×), and 6 μL ddH2O. The reaction conditions included 40 cycles of pre-denaturation at 95 °C for 30 s, denaturation at 95 °C for 5 s, annealing at 62°C for 34 s, and an extension at 95 °C for 15 s. Maize *actin 1* was used as the internal reference gene to normalize the gene expression data. Eight DEPs were selected to evaluate the consistency of the protein expression level and the mRNA expression level. Primer Premier 5 software (Premier Biosoft International, CA, USA) was used to design primers by finding the corresponding gene sequences in the gene database (Table S10). The relative expression levels were calculated using the 2^−∆∆Ct^ method. Three biological replicates were set for each treatment.

### 4.7. Bioinformatics Analysis

SPSS 19.0 statistical software (SPSS Inc., Armonk, NY, USA) was used to analyze the data, and Duncan’s test was used to assess the significant differences between different treatments. The experimental results were expressed as the mean ± standard deviation, and three biological replicates were tested. Microsoft Excel was used for mapping.

The raw mass spectral data were converted to mgf format, and the final protein identification was obtained by comparison with sequences in the Mascot search engine (Matrix Science, London, UK; version 2.3.02) and the Uniprot *Zea mays* L. database (140,026 sequences, 2017_03_24). Protein identification had a mass tolerance of 0.05 Da for intact peptides and 0.1 Da for fragmented peptides, allowing for one missed cleavage in the trypsin digests. Settings included Oxidation (M), iTRAQ8plex (Y) as the potential variables, and Carbamidomethyl (C), iTRAQ8plex (N-term), iTRAQ8plex (K) were used as the fixed modifications. Only peptides at the 95% confidence interval were counted as identified. In addition, each confident protein identification involved at least one unique peptide. The MS-based proteomic data are available via ProteomeXchange with identifier PXD012344. For protein quantitation, it was required that a protein be represented by at least two unique spectra. The control samples were used as a reference, based on the weighted average of the intensity of reported ions in identified peptides. The quantitative protein ratios were weighted and normalized to the median ratio in Mascot. A protein was considered statistically significant only when *p* values < 0.05, and a fold change of > 1.20 or < 0.83 in at least one two biological replicates.

The identified proteins were categorized according to their GO annotation (http://www.geneontology.org/, accessed on 28 March 2017), and the hypergeometric test was used to find significant enrichment GO entries by comparing significantly different proteins with the overall identified proteins as background. If the hypergeometric test had a *p* < 0.05, then the DEPs were significantly enriched in the GO terms. The COG analysis was also conducted (http://www.ncbi.nlm.nih.gov/COG/, accessed on 28 March 2017). The metabolic pathway analysis of the proteins was conducted according to the Kyoto Encyclopedia of Genes and Genomes (KEGG) Pathway Database (http://www.genome.jp/kegg, accessed on 28 March 2017r).

## 5. Conclusions

In this study, we used an iTRAQ quantitative approach coupled with LC-MS/MS and bioinformatics to identify the maize seedling root proteins of the drought-tolerant Chang 7-2 and drought-sensitive TS141 under drought stress. The response mechanism of Chang 7-2 to drought was attributed to: The stronger water retention capacity; the elimination of harmful free radicals and alleviation of oxidative stress via the synergistic action of antioxidant enzymes, such as POD; the strengthen of cell wall by xyloglucan endotransglucosylase/hydrolase; and the reduction in the extravasation of cellular contents, maintenance of osmotic balance, and stabilization of the membrane system of plants by the acyl-CoA-binding protein, lipid binding protein, and asparagine synthetase. Besides, the increase in ubiquitin fusion degradation protein made Chang 7-2 more efficient in recycling amino acids from proteins that have been inactivated by drought exposure. Furthermore, the reduction in cytosolic aldehyde dehydrogenase RF2D improved the lignification degree. In addition, the two varieties were found to have a common mechanism under stressed conditions: The production of more energy through the glycolysis/gluconeogenesis pathway and related enzymes; the alleviation of the toxic effects of aldehydes and ammonia by aldehyde dehydrogenase, alcohol dehydrogenase and glutamate synthase; and maintain cell membrane stability through the effective of s-adenosylhomocysteine hydrolase and non-symbiotic hemoglobin.Some enzymes also responded to drought stress by regulating respiration and increasing the ethylene content.

## Figures and Tables

**Table 1:** Significant enrichment analysis of DEP Pathways in CD vs. CCK (*p* < 0.05).

Pathway	Number of DEPs(1007)	*p*-Value	Pathway ID
Ribosome	54	0.006156876	ko03010
Glycosphingolipid biosynthesis—globo and isoglobo series	5	0.006714952	ko00603
Nitrogen metabolism	11	0.009075108	ko00910
Amino sugar and nucleotide sugar metabolism	31	0.009913536	ko00520
Pentose phosphate pathway	17	0.02244357	ko00030
Mismatch repair	8	0.0247802	ko03430
Fructose and mannose metabolism	19	0.02541227	ko00051
Glycolysis/Gluconeogenesis	33	0.02772382	ko00010
Glycosphingolipid biosynthesis—ganglio series	4	0.03880259	ko00604
Phagosome	17	0.0488682	ko04145

**Table 2:** Significant enrichment analysis of DEP Pathway in TD vs. TCK (*p* < 0.05).

Pathway	Number of DEPs(350)	*p*-Value	Pathway ID
RNA polymerase	10	0.000241549	ko03020
Metabolic pathways	125	0.000448353	ko01100
Carbon fixation in photosynthetic organisms	12	0.001034286	ko00710
Nitrogen metabolism	6	0.007834169	ko00910
Glycerophospholipid metabolism	9	0.008846003	ko00564
Starch and sucrose metabolism	15	0.01968736	ko00500
Amino sugar and nucleotide sugar metabolism	13	0.02511713	ko00520
Glycolysis/Gluconeogenesis	14	0.0361396	ko00010
Galactose metabolism	8	0.04039815	ko00052
Phenylpropanoid biosynthesis	18	0.04056657	ko00940
Alanine, aspartate and glutamate metabolism	7	0.0461047	ko00250

## Supplementary Materials

Supplementary materials can be found at https://www.mdpi.com/1422-0067/20/11/2793/s1. Table S1. All proteins identified by iTRAQ in maize seedling roots, Table S2. The peptide information of all proteins, Table S3. The identified spectra, Table S4. Proteins up-regulated in Chang 7-2, Table S5. Proteins down-regulated in Chang 7-2, Table S6. Proteins up-regulated in TS141, Table S7. Proteins down-regulated in TS141, Table S8. Proteins up-regulated in Chang 7-2 and TS141, Table S9. Proteins with different direction of accumulation, Table S10. Primers used for qRT-PCR analysis, Figure S1. Phenylpropanoid biosynthesis pathway. (A) CD vs. CCK. (B) TD vs. TCK.

## Abbreviations

iTRAQ	Isobaric Tags for Relative and Absolute Quantitation
PEG6000	Polyethylene glycol 6000
DEPs	Differentially Expressed Proteins
qRT-PCR	Quantitative real-time PCR
2-DE	Two-Dimensional Electrophoresis
CK	Control
D	Drought Stress
CCK	Chang 7-2 Control
CD	Chang 7-2 Drought Stress
TCK	TS141 Control
TD	TS141 Drought Stress.
SL	Seeding Length
RL	Root Length
SFW	Seedling Fresh Weight
RFW	Root Fresh Weight
RWC	Relative Water Content
REL	Relative Electrolyte Leakage
MDA	Malondialdehyde
Pro	Proline
SOD	Superoxide Dismutase
POD	Peroxidase
CAT	Catalase
COG	Cluster of Orthologous Groups of Proteins
GO	Gene Ontology
UFD	Ubiquitin Fusion Degradation Protein
ROS	Reactive Oxygen Species
CA	Coniferyl Aldehyde
SA	Sinapaldehyde
PFP	Pyrophosphate: Fructose-6-Phosphate 1-Phosphotransferase
PFK	Phosphofructokinase
PPi	Pyrophosphoric acid
PK	Pyruvate Kinase
GAPDH	Glyceraldehyde 3-Phosphate Dehydrogenase
PDC	Pyruvate Decarboxylase
ACO	1-Amino-Cyclopropane-1-Carboxylate Oxidase
ACC	1-Aminocyclopropane-1-Carboxylate
AlaAT	Alanine Aminotransferase
LC-MS/MS	Liquid Chromatography-Electrospray Ionization-Tandem Mass Spectrometry
KEGG	Kyoto Encyclopedia of Genes and Genomes
