# Supplementary material for: Comparative Proteomics Analysis of the Seedling Root Response of Drought-sensitive and Drought-tolerant Maize Varieties to Drought Stress
Source: Int J Mol Sci. 2019 Jun 7;20(11):2793. doi: 10.3390/ijms20112793 (PMC6600177; doi:10.3390/ijms20112793)
Supplement: Supplementary file 1 [file Figure S1.pdf › Supplementary Materials/Figure S1.pdf]

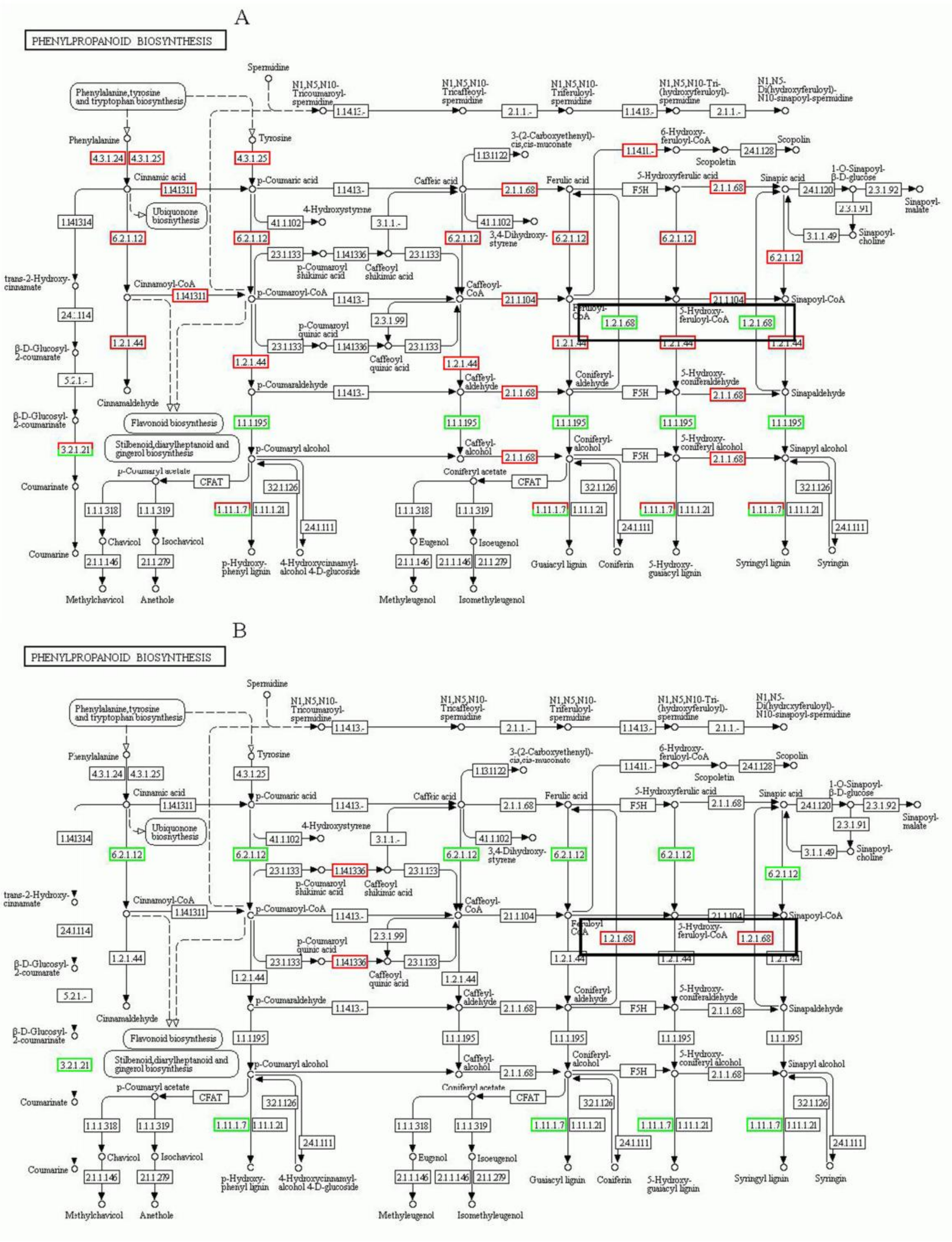

Figure S1. Phenylpropanoid biosynthesis pathway. (A) CD vs. CCK. (B) TD vs. TCK.
